# Accumulation of Biological and Behavioral Data of Female Sex Workers Using Respondent-Driven Sampling: Protocol for a Systematic Review

**DOI:** 10.2196/43722

**Published:** 2023-06-19

**Authors:** Mihir Bhatta, Agniva Majumdar, Sitikantha Banerjee, Piyali Ghosh, Subrata Biswas, Shanta Dutta

**Affiliations:** 1 National Institute of Cholera and Enteric Diseases Kolkata India

**Keywords:** respondent-driven sampling, RDS, female sex worker, FSW, systematic review, review method, sex worker, sampling method, sample design

## Abstract

**Background:**

Respondent-driven sampling (RDS) is a nonprobability sampling technique that allows the extrapolation of its outcome to the target population. This approach is typically used to overcome the difficulties in studying hidden or difficult-to-reach groups.

**Objective:**

The purpose of this protocol is to generate a systematic review on the accumulation of biological and behavioral data of female sex workers (FSWs) through different surveys that use the RDS method from around the world in the near future. The future systematic review will discuss the initiation, actualization, and problems of RDS during the accumulation of biological and behavioral data of FSWs through surveys from around the world.

**Methods:**

The behavior and biological data of FSWs will be extracted from peer-reviewed studies published between 2010 and 2022 and that are acquired through RDS. Using PubMed, Google Scholar, the Cochrane database, Scopus, Science Direct, and the Global Health network, all papers that are available will be obtained using the search phrases “respondent-driven” and “Female Sex Workers” OR “FSW” OR “sex workers” OR “SW.” According to STROBE-RDS (Strengthening the Reporting of Observational Studies in Epidemiology for Respondent-Driven Sampling) criteria, the data will be retrieved through a data extraction form and will be organized using World Health Organization classifications of areas. The Newcastle-Ottawa Quality Assessment Scale will be used to measure bias risk and overall study quality.

**Results:**

The future systematic review that will be generated from this protocol will offer evidence for or against the claim that using the RDS technique to recruit participants from “hidden” or “hard-to-reach” populations is the best strategy. The results will be disseminated through a peer-reviewed publication. Data collection started on April 1, 2023, and the systematic review is expected to be published by December 15, 2023.

**Conclusions:**

A minimum set of parameters for specific methodological, analytical, and testing procedures, including RDS methods to evaluate the overall quality of any RDS survey, will be provided by the future systematic review, in accordance with this protocol, to assist researchers, policy makers, and service providers in improving RDS methods for the surveillance of any key population.

**Trial Registration:**

PROSPERO CRD42022346470; https://tinyurl.com/54xe2s3k

**International Registered Report Identifier (IRRID):**

DERR1-10.2196/43722

## Introduction

### Background

The respondent-driven sampling (RDS) method is a nonprobability sampling method that approximates probability sample design, allowing for the extrapolation of results to the target population. This method is generally used to address the limitations of studying hidden or hard-to-reach populations [1]. The term “hard-to-reach population” emerged in the early 1990s in public health to refer to the comparatively lower socioeconomic and moderate to low literacy groups, ethnic minority groups, and those who are not successfully reached by health workers from different health campaigns. Social science workers coined the term “hidden population” to refer to the group of people with an inadequate sampling frame. This situation can occur when they belong to an unorthodox occupational group (female sex workers [FSWs]) or closed social groups (men who have sex with men) or have outlawed behaviors (people who inject drugs) or small population size, among others [1].

Initially, RDS was developed [2] as a chain-reference sampling technique by which a data-tracing path is obtained from one person to another, based on their relationship consecutively. This method combines aspects from snowball sampling, stochastic Markov chain modelling, and the theory of biased networks (homophily model) [1]. The authors also rely on the theory describing the “small world” phenomenon that each person indirectly associates with another person through approximately 6 intermediaries, no matter how large the network. If this premise is true, it would mean that even the most socially isolated individuals can be reached in the sixth wave of a reference chain, starting from any arbitrarily chosen individual [2]. Other theoretical bases for the “small world” phenomenon come from sociology—particularly from behavioral theories that study obedience or social control based on the group of belonging [2]. This theoretical background justifies the idea of incentive and peer involvement in recruitment [2].

RDS has been used in several countries to collect data from populations at high risk of HIV exposure, FSWs, men who have sex with men, people who use or inject drugs, and other such people considered “hard-to-reach” due to social stigma and the exercise of unsocial activities [1,3]. Over the decades, this widely accepted sampling technique is used, with the recommendations of organizations such as the US Centers for Disease Control and Prevention, the Joint United Nations Programme on HIV/AIDS (UNAIDS), the World Health Organization (WHO) Global Fund, and others, to generate data on the baseline, conduct trend analysis for prevalence estimation, and study risk behaviors and the impact of the program on HIV and other sexually transmitted infections through biological and behavioral assessments [3]. The sampling technique has proved feasible and successful in recruiting hidden populations of people who inject drugs in a variety of settings, resulting in the rapid acquisition of long and varied recruitment chains. It has been used in several countries to collect both behavioral and biological data from sex workers (eg, Vietnam and India) and men who have sex with men (eg, Bangladesh, Cambodia, Uganda, and the United States) [3,4]. RDS starts with a fixed number of participants called “seeds,” which are carefully chosen by the surveillance team from the target population, and samples are built through the passing of a coupon from one peer to the next. A small number of coupons are generated for each participant, limiting the overcontribution of participants with a greater number of ties to other people in the same network. These coupons allow researchers to observe the procedure of recruitment by limiting participants from having to deliver individual information about their recruits [5]. Individuals who are participating and recruiting peers for the survey are provided with “incentives” to ensure ongoing participation and proper recruitment. This participation and recruitment process may result in a long recruitment chain made up of plentiful “waves” of recruits. The arrangement of the sample becomes less reliant on the purposefully selected seeds as recruitment chains get elongated [6]. Statistical modifications for variation in network sizes and recruitment efforts are used after the sample collection to generate data on estimates that are representative of the population’s network [1,2,5].

FSWs are also hard to reach because they are mostly mobile and regularly change solicitation points—districts and towns within and across states or provinces [7-9]. Some FSWs are difficult to locate as they engage in part-time sex work. Higher-paid sex workers, such as those who solicit through the internet and agents, have the capacity to remain hidden [8-10]. However, previous involvement with RDS is limited and needs further evaluations to validate these methods. Furthermore, little is known about the feasibility of this sampling technique as a method of recruiting hidden populations of FSWs in settings where the organization of sex work and access to FSWs is highly controlled and where there is little or no contact between the target population and local services [7,11].

### Objective

In the context of this background, the purpose of this protocol is to generate a systematic review on the accumulation of biological and behavioral data of FSWs through different surveys that use the RDS method from around the world in the near future. The future systematic review will discuss the initiation, actualization, and problems of RDS during the accumulation of biological and behavioral data of FSWs through surveys from around the world.

## Methods

### Overview

The protocol has been developed in accordance with the STROBE-RDS (Strengthening the Reporting of Observational Studies in Epidemiology for Respondent-Driven Sampling) guideline (Multimedia Appendix 1) [11]. This protocol has been registered in PROSPERO (CRD42022346470).

### Review Items

According to the STROBE-RDS [12] guideline, the following items will be captured in the review:

1. Author
2. Year of publication along with period of study
3. Country or geographical location of study
4. Presurvey assessment (if any)
5. Number of RDS data collection sites
6. Interview methods
7. Numbers of initial and final seeds along with the maximum number of waves
8. Amount of primary and secondary incentives
9. The target sample size and sample size obtained finally
10. Design effect used in sample size calculation
11. The duration of data collection (in weeks)
12. Maximum number of coupons distributed to each recruiter
13. Whether equilibrium or convergence was assessed or not
14. Whether the data were adjusted for network size
15. Use of software for statistical analysis

### Electronic Database and Search Terms

All available articles will be retrieved using PubMed, Google Scholar, the Cochrane database, Scopus, Science Direct, and the Global Health network. Search terms will include “respondent-driven” OR “RDS” and “Female Sex Workers” OR “FSW” OR “sex workers” OR “SW.” The full search terms are presented in Multimedia Appendix 2.

### Inclusion Criteria

The inclusion criteria are as follows:

1. Peer-reviewed literature published only in English in a physical or web-based format that reported using RDS
2. Article published from January 2010 to December 2022
3. Articles on FSWs only

FSWs are defined as those who are cisgender female and have ever exchanged sex for money or goods within the last 12 months.

### Exclusion Criteria

Duplicates, irrelevant articles (eg, protocols, presentations, flyers, and those not strictly addressing RDS methodology) along with reviews, opinion pieces, editorials, commentaries, and studies that are not intended to report population-based estimates will be excluded from the initial extraction.

### Selection of Eligible Studies

We will obtain the full text of articles or other documents reporting studies identified as being potentially eligible for inclusion. Two suitably qualified reviewers will screen titles and abstracts of articles identified by the search strategy independently using the data extraction form (Multimedia Appendix 3). Any study selected as being potentially eligible by either reviewer will be retained for review of the full text. If no abstract is available electronically and eligibility cannot be judged from the title alone, the full text of the article will be retrieved and screened. The abstracts of articles identified through additional searches will be reviewed in the same manner as those identified through database searches.

### Strategy for Data Synthesis

The data will be extracted from full-text published articles through Epi Inf (version 6.0) [13] using a modified data extraction form (Multimedia Appendix 3) according to the STROBE-RDS guideline.

The data will be arranged into 6 subtables in a Microsoft Excel spreadsheet based on WHO classifications of regions [14], such as the African Region, Region of the Americas, South-East Asian Region, European Region, Eastern Mediterranean Region, and Western Pacific Region.

### Deduplication

Mendeley bibliographic software [15] will be used for reference management. The following rules will be used to remove duplicate hits from the database:

1. Compare the title or various combinations of the author, year, secondary title, volume, issue, and pages through “deduplication”;
2. Visually compare the full records of suspected duplicates; and
3. Save duplicates in a separate database.

### Risk of Bias and Quality Assessment

The evaluation of articles through the title, abstract, and entire text of the manuscripts will be performed prior to the addition of it to the ultimate analysis. Assessment will be performed with the help of the Newcastle-Ottawa Quality Assessment Scale [16].

### Ethical Consideration

Formal ethical approval is not required as primary data will not be collected.

## Results

The future systematic review that will be generated from this protocol will offer evidence for or against the claim that using the RDS technique to recruit participants from “hidden” or “hard-to-reach” populations is the best strategy. The results will be disseminated through a peer-reviewed publication. Data collection started on April 1, 2023, and the systematic review is expected to be published by December 15, 2023.

## Discussion

A systematic review will be generated from this protocol and will provide evidence in support of or against the hypothesis that the application of the RDS technique is an effective approach to recruiting participants from “hidden” or “hard-to-reach” populations. This peer-to-peer recruitment is driven by legitimate coupons, making it successful among populations that are stigmatized or who practice behaviors that are considered illegal in the existing social structure.

Similar to any systematic review protocol, this protocol and the future review will also be restricted by the comprehensiveness of the published articles and whether researcher publish their study in open access and peer-reviewed journals. Moreover, the included articles on surveys that accumulate biological data would lead to further studies.

As sex work also carries occupational risk, this approach is expected to be appropriate and the best suited for this high-risk population [17]. The future systematic review, as conducted according to this protocol, will help researchers, policy makers, and service providers to improve the RDS methods for the surveillance of any key population, by providing a minimum set of parameters of specific methodological, analytical, and testing procedures, including RDS methods to evaluate the overall quality of any RDS survey [18].

## Appendix

Multimedia Appendix 1 STROBE-RDS (Strengthening the Reporting of Observational Studies in Epidemiology for Respondent-Driven Sampling) study reporting checklist.

Multimedia Appendix 2 Search terms.

Multimedia Appendix 3 Data extraction form.

## Abbreviations

FSW: female sex worker
RDS: respondent-driven sampling
MSM: male sex with male
STROBE-RDS: Strengthening the Reporting of Observational Studies in Epidemiology for Respondent-Driven Sampling
UNAIDS: United Nations Programme on HIV/AIDS
WHO: World Health Organization
