# Supplementary material for: Accumulation of Biological and Behavioral Data of Female Sex Workers Using Respondent-Driven Sampling: Protocol for a Systematic Review
Source: JMIR Res Protoc. 2023 Jun 19;12:e43722. doi: 10.2196/43722 (PMC10337474; doi:10.2196/43722)
Supplement: Multimedia Appendix 1 [file resprot_v12i1e43722_app1.docx]

*Multimedia Appendix 1:* **STROBE-RDS study reporting checklist**

Guideline development proceeding according to *Moher et al* 2010. This checklist is adapted from STROBE guidelines. The checklist scope is limited to RDS reports that seek to generate representative estimates for populations or risk factor studies, as these are currently the most contentious and potentially most policy-relevant uses of RDS.

|  | **Item** | **Recommendation** |
| --- | --- | --- |
| **Title and abstract** | 1 | a) Indicate the study’s design (Respondent-Driven Sampling) in the title or abstract |
|  |  | b) Provide in the abstract an informative and balanced summary of what was done and what was found |
| **Introduction** |  |  |
| Background/rationale | 2 | Explain the scientific background and rationale for the investigation being reported |
| Objectives | 3 | State specific objectives, including any pre-specified hypotheses |
| **Methods** |  |  |
| Study design | 4 | a) Present key elements of study design early in the paper |
|  |  | b) State why RDS considered the most appropriate sampling method |
| Setting | 5 | a) Describe the setting, location(s), and relevant dates, including periods of recruitment  and data collection. If a risk factor study, also describe exposures |
|  |  | b) Describe formative research methods & findings used to inform RDS study design |
| Participants | 6 | a) Give the eligibility criteria, number, sources and methods of seed selection |
|  |  | b) State if additional seeds were required, and if so, when and how recruited and started |
|  |  | c) State if there was any variation in study design during data collection (eg changing numbers of coupons per recruit, or stopping chains) |
|  |  | d) Give the eligibility criteria for subsequent recruits if it differs from seeds |
|  |  | e) Give number, types (eg mobile/static) & location of recruitment venue(s) |
|  |  | f) Consider reporting information on coupons(s). |
|  |  | g) Report wording of network size question(s) |
|  |  | h) Describe how participants were trained/ instructed to recruit others, including  maximum number of recruitments, any maximum time referral permitted, and any efforts to encourage random sampling within recruits’ network |
|  |  | i) Refer to ethical review clearance documents |
|  |  | j) Consider reporting recruitment challenges (eg selling of coupons, imposters, duplicate recruits) |
| Variables | 7 | a) Clearly define all outcomes, and if applicable exposures, predictors, potential  confounders, and effect modifiers. Give diagnostic criteria, if applicable |
|  |  | b) State if and how recruiter-recruit relationship was tracked |
|  |  | c) Consider reporting additional social network data, if available |
|  |  | d) Consider reporting information on composition of personal networks, if available |
| Data sources/ measurement | 8* | a) For each variable of interest, give sources of data (eg instrument) and details of methods of assessment (measurement). Describe comparability of assessment methods  if there is more than one group |
|  |  | b) Describe incentives for participation and recruitment |
|  |  | c) Describe methods to assess eligibility and reduce repeat enrollment (eg coupon manager software, biometrics, detection of commercial exchange of coupons) |

|  |  | d) QA/C checks (eg were returned coupons actually distributed & redeemed only once?) |
| --- | --- | --- |
| Bias | 9 | Describe any efforts to address potential sources of bias |
| Study size | 10 | Explain how the sample size was arrived at |
| Quantitative variables | 11 | Explain how quantitative variables were handled in the analyses. If applicable, describe which groupings were chosen and why |
| Statistical methods | 12 | a) Describe all statistical methods, including name and description of the analytical methods (ie point and interval estimators) used to take account of RDS sampling strategy. If appropriate, report software package used with version number and settings  values |
|  |  | b) Report any criteria used to support statements on whether estimator conditions or assumptions were met eg ‘RDS equilibrium reached’ |
|  |  | c) State if seeds included in each analysis |
|  |  | d) If applicable, describe methods used to control for confounding |
|  |  | e) Describe any methods used to examine subgroups and interactions |
|  |  | f) Explain how missing data and small numbers were addressed |
|  |  | g) Describe any sensitivity analyses |
| **Results** |  |  |
| Participants | 13* | a) Report numbers of individuals at each stage of study, ie final number of seeds, number examined for eligibility, number confirmed eligible, number included in study, number returned for incentive collection and (if applicable) re-interview, and number  analysed. Consider use of a flow diagram to summarise this. |
|  |  | b) Give reasons for non-participation at each stage, including if data collected reported reason for coupon rejection |
|  |  | c) Report number of coupons distributed and returned |
|  |  | d) Report number of recruits by seed and number of RDS recruitment waves. Consider showing graph of entire recruitment network, whilst ensuring anonymity maintained. |
|  |  | e) Consider reporting numbers potentially eligible if population size estimates made |
| Descriptive data | 14* | a) Give characteristics of study participants (eg demographic, clinical, social) if  appropriate, information on exposures and potential confounders |
|  |  | b) Indicate number of participants with missing data for each variable of interest |
| Outcome data | 15* | Report numbers of outcome events or summary measures |
| Main results | 16 | a) Report unadjusted estimates and their stated precision (eg, 95% confidence interval) |
|  |  | b) If applicable, report adjusted estimates and their stated precision (eg, 95% CI) |
|  |  | c) If adjusted estimates presented, report enough information so that the reason for the magnitude of the adjustment is clear (eg network sizes and homophily by group) |
|  |  | d) If appropriate, make clear which confounders were adjusted for and why included |
|  |  | e) Report category boundaries when continuous variables were categorized |
|  |  | f) If relevant, consider translating estimates of relative risk into absolute risk for a meaningful time period |

Other analyses 17 Report other analyses done—eg

- 1. analyses of subgroups and interactions
  2. sensitivity analyses eg different RDS estimators, different definitions of network size

### Discussion

Key results 18 Summarise key results with reference to study objectives

Limitations 19 Discuss limitations of the study, taking into account sources of potential bias or imprecision. Consider the limitations of cross-sectional studies, the RDS sampling method and, if used the RDS method(s) of inference. Include comment on how representative the unadjusted sample is thought to be. Indicate how participants compare to population description developed during formative assessment and other sources of information. Discuss both direction and magnitude of any potential bias

Interpretation 20 Give a cautious overall interpretation of results considering objectives, limitations, multiplicity of analyses, results from similar studies, and other relevant evidence

Generalisability 21 Discuss the generalisability (external validity) of the study results

### Other information

Funding 22 Give the source of funding and the role of the funders for the present study and, if applicable, for the original study on which the present article is based

Data sharing 23 State whether access provided to data and survey, and if so, how to access
