# Supplementary material for: Accumulation of Biological and Behavioral Data of Female Sex Workers Using Respondent-Driven Sampling: Protocol for a Systematic Review
Source: JMIR Res Protoc. 2023 Jun 19;12:e43722. doi: 10.2196/43722 (PMC10337474; doi:10.2196/43722)
Supplement: Multimedia Appendix 2 [file resprot_v12i1e43722_app2.docx]

***Multimedia Appendix 2: Search terms***

**(respondent-driven) OR (respondent-driven sampling) OR (respondent-driven survey) OR RDS**

(("respondent-driven"[All Fields] OR ("respondent-driven"[All Fields] OR ("sample"[All Fields] OR "sample s"[All Fields] OR "sampled"[All Fields] OR "samples"[All Fields] OR "sampling"[All Fields] OR "samplings"[All Fields])) OR ("respondent-driven"[All Fields] OR ("surveys"[All Fields] OR "surveyed"[All Fields] OR "surveying"[All Fields] OR "surveys OR questionnaires"[MeSH Terms] OR ("surveys"[All Fields] OR "questionnaires"[All Fields]) OR "surveys OR questionnaires"[All Fields] OR "survey"[All Fields] OR "surveys"[All Fields]))

**AND**

**(Female Sex workers) OR FSW or (sex workers) OR SW**

((("female"[MeSH Terms] OR "female"[All Fields] OR ("female"[All Fields] OR sex"[All Fields]) OR "female sex"[All Fields]) OR ("occupational groups"[MeSH Terms] OR ("occupational"[All Fields] OR "groups"[All Fields]) OR "occupational groups"[All Fields] OR "worker"[All Fields] OR "workers"[All Fields] OR "worker s"[All Fields])) OR "FSW"[All Fields] OR ("sex workers"[MeSH Terms] OR "sex workers"[All Fields]) OR "SW"[All Fields])
