# Supplementary figures and images for: Accumulation of Biological and Behavioral Data of Female Sex Workers Using Respondent-Driven Sampling: Protocol for a Systematic Review
Source: JMIR Res Protoc. 2023 Jun 19;12:e43722. doi: 10.2196/43722 (PMC10337474; doi:10.2196/43722)

***Multimedia Appendix 3: Data extraction form***
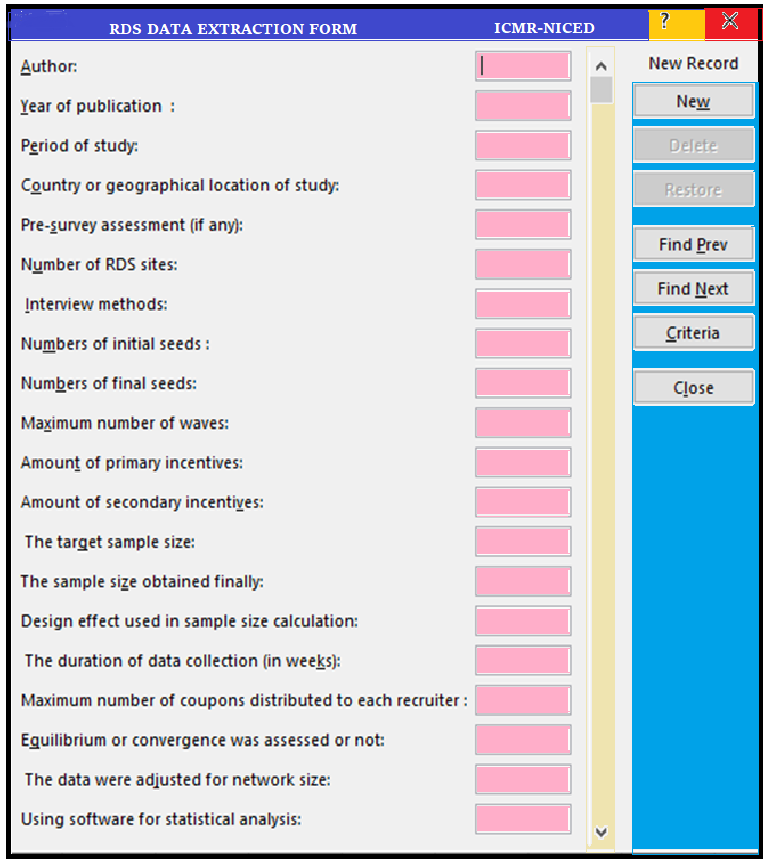

Supplement: Multimedia Appendix 3 [file resprot_v12i1e43722_app3.docx]
